# Renal allograft rejection, lymphocyte infiltration, and de novo donor-specific antibodies in a novel model of non-adherence to immunosuppressive therapy

**DOI:** 10.1186/s12865-017-0236-6

**Published:** 2017-12-19

**Authors:** Louisa Kühne, Bettina Jung, Helen Poth, Antonia Schuster, Simone Wurm, Petra Ruemmele, Bernhard Banas, Tobias Bergler

**Affiliations:** 1 Department of Nephrology, University Hospital Regensburg, Franz-Josef-Strauß Allee 11, D-93053 Regensburg, Germany; 2 Department of Pathology, University Hospital Erlangen, Erlangen, Germany

**Keywords:** Donor specific antibodies, Humoral rejection, Renal transplantation, Non-adherence, Leukocyte infiltration, BAFF

## Abstract

**Background:**

Non-adherence has been associated with reduced graft survival. The aim of this study was to investigate the immunological mechanisms underlying chronic renal allograft rejection using a model of non-adherence to immunosuppressive therapy. We used a MHC (major histocompatibility complex) -mismatched rat model of renal transplantation (Brown Norway to Lewis), in which rats received daily oral cyclosporine A. In analogy to non-adherence to therapy, one group received cyclosporine A on alternating days only. Rejection was histologically graded according to the Banff classification. We quantified fibrosis by trichrome staining and intra-graft infiltration of T cells, B cells, and monocytes/macrophages by immunohistochemistry. The distribution of B lymphocytes was assessed using immunofluorescence microscopy. Intra-graft chemokine, chemokine receptor, BAFF (B cell activating factor belonging to the TNF family), and immunoglobulin G transcription levels were analysed by RT-PCR. Finally, we evaluated donor-specific antibodies (DSA) and complement-dependent cytotoxicity using flow cytometry.

**Results:**

After 28 days, cellular rejection occurred during non-adherence in 5/6 animals, mixed with humoral rejection in 3/6 animals. After non-adherence, the number of T lymphocytes were elevated compared to daily immunosuppression. Monocyte numbers declined over time. Accordingly, lymphocyte chemokine transcription was significantly increased in the graft, as was the transcription of BAFF, BAFF receptor, and Immunoglobulin G. Donor specific antibodies were elevated in non-adherence, but did not induce complement-dependent cytotoxicity.

**Conclusion:**

Cellular and humoral rejection, lymphocyte infiltration, and de novo DSA are induced in this model of non-adherence.

**Electronic supplementary material:**

The online version of this article (doi: 10.1186/s12865-017-0236-6) contains supplementary material, which is available to authorized users.

## Background

Although short-term renal allograft survival has improved over the past decades, long-term graft survival is still limited with overall 5- and 10-year graft survival rates of 77 and 56% respectively in Europe [1]. Research efforts have therefore focused on identifying ways to prolong graft survival. A multitude of factors are responsible for chronic allograft failure, including concomitant disease, calcineurin-inhibitor (CNI) toxicity, recurrent or de novo renal disease, as well as chronic allograft rejection [2]. Among these factors, chronic rejection most frequently causes graft failure [2, 3]. In this context, subclinical inflammation in renal allografts [4–6] and the serological appearance of de novo donor-specific antibodies (*dn*DSA) have been strongly implicated as factors for reduced graft survival [3, 7–9]. A major cause for the formation of de novo DSA is non-adherence to immunosuppressive therapy [3], and numerous studies show that non-adherence itself is a risk factor for reduced graft survival [10–12].

Antibody mediated rejection (ABMR) is known to be a major contributor to graft loss [3]. Histologically, the hallmark feature of chronic antibody mediated rejection is transplant glomerulopathy (TG), which has been shown to correlate with the formation of DSA and specific patterns of C4d deposition [13]. Interstitial fibrosis and tubular atrophy (IF/TA), though non-specific, frequently accompany TG in chronic ABMR. Another histopathological feature, which has been observed in chronically rejected grafts, are B cell rich tertiary lymphoid organs (TLO) [14], which have also been found in other types of chronically inflamed tissues.

Since *dn*DSA and ABMR are prominent causes of graft failure, increasing attention has been drawn to B cells, due to their function as antibody-producers, as well as regulatory functions, such as cytokine production. However, so far B cells have not been specifically targeted by standard immunosuppressive protocols in renal transplantation, apart from some special applications, such as ABO-incompatible renal transplantation. Furthermore, the benefit of B cell depleting agents, such as Rituximab, in the treatment of ABMR remains controversial [15]. Other B cell targeted therapies have been developed for chronic inflammatory and autoimmune diseases [16, 17], but their contribution in allogeneic solid organ transplantation is still under scrutiny.

Previously, we reported on a notable difference in the relative infiltration of B-lymphocytes in the context of allogeneic vs. syngeneic transplantation in a rat kidney transplant model [18]. In the current study, we used a rat model reflecting non-adherence to immunosuppressive therapy, to answer to following questions: 1.) to what extent can non-adherence cause rejection 2.) what effect does non-adherence have on infiltrating leukocyte populations 3.) how are chemokine transcription patterns affected 4.) how are intra-renal B-cells affected and 5.) does non-adherence result in the development of donor specific antibodies?

To this end, we used a MHC-mismatched rat model of allogeneic renal transplantation.

## Methods

### Animals/experimental renal transplantation

Animal experiments were performed according to German animal protection laws and NIH’s laboratory animal care principles. Study approval was granted by the inspecting authority (Regierung der Oberpfalz). A MHC-mismatched rat kidney transplantation model was used, as previously described [19]. Male Brown Norway rats (BN) served as donors and male Lewis rats (LEW) as recipients (Charles River Laboratories, Sulzfeld, Germany, 200–250 g). Kidney transplantation was either syngeneic (Lewis-to-Lewis) or allogeneic (Brown-Norway-to-Lewis).

Kidney transplantation (Tx) was performed as previously described [19]. In brief, left BN kidneys were explanted, flushed with cold saline and transplanted orthotopically in Lewis rats by end-to-end anastomosis of the ureter and blood vessels. Cold and warm ischemia times were approximately 35 and 30 min, respectively. Nephrectomy of the right kidney was performed at the end of the surgery.

All animals with allogeneic transplantation were treated with cyclosporine A (CyA 5 mg/kg body weight; Neoral, Novartis, Basel, Switzerland), administered once daily by gavage. In one group, CyA was administered only on alternating days after day 6, in analogy to non-adherence to immunosuppressive therapy (“Tx d28 CyA alt.”). Rats were sacrificed 6 and 28 days after transplantation. In general, syngeneic transplantation was used as a control, except for complement-dependent-cytotoxicity assay, where Lewis rat serum from day 0 was used.

Groups were abbreviated as follows:

Syngeneic transplantation sacrificed on day 6 (SynTxd6), allogeneic transplantation with daily CyA sacrificed on day 6 or day 28 (Txd6CyA, Txd28CyA), and allogeneic transplantation with CyA administered on alternating days, sacrificed on day 28 (Txd28CyAalt). Groups consisted of 6 to 11 animals.

Harvested organs were divided into quarters and either fixed in paraffin or snap-frozen in N_2_ and stored at −80 °C, or processed for flow cytometry.

### Histology, Immunohistochemistry and Immunofluorescence

Paraffin sections were prepared from rat kidneys as previously described [20]. After staining with hematoxylin and eosin (HE) and periodic acid schiff (PAS) stains, the histomorphological alterations were classified according to the Banff classification [21] by an experienced pathologist.

Immunohistochemistry was performed on 3 μm formalin-fixed, paraffin-embedded sections as previously described [18]. Primary antibodies included polyclonal rabbit anti-rat CD3 antibody (1:100, Abcam, ab5690, Cambridge, UK), polyclonal goat anti-rat CD20 antibody (1:100, Santa Cruz, sc-7735, Heidelberg, Germany), monoclonal mouse anti-rat CD68 antibody (1:150, Serotec, MCA341GA, Oxford, UK), rabbit anti-rat C4d antibody (Hycultec, HP8034, Beutelsbach, Germany), goat polyclonal anti-CCL21 / SLC antibody (aa24–133) (LS-C150160, Lifespan Biosciences, Seattle, USA), rabbit monoclonal anti-CCR7 antibody (Y59, ab32527, Abcam, Cambridge, UK). Secondary antibodies were goat anti-rabbit-biotin (Dianova, 111–065-144, Hamburg, Germany), mouse anti-goat-biotin (Dianova, 205–065-108, Hamburg, Germany), donkey anti-mouse-biotin (Dianova, 715–065-150, Hamburg, Germany). Staining was done using DAB (0.4 mg/ml, Sigma, D5637, St. Louis, USA) and AP-RED (Zytomed, ZUC001–125, Berlin, Germany). For CCL21 staining, anti-goat-Polymer-HRP Kit (Vector laboratories, Immpress Reagent Anti-Goat Ig HRP, MP-7405, Peterborough, UK) was used as a secondary antibody. C4d staining was enhanced using AP-One-Step Polymer (Zytomed, ZUC068–006, Berlin, Germany) with Permanent AP RED Kit (Zytomed, ZUC001–125, Berlin, Germany) as secondary antibody. For CCL21 and CCR7, two to three sections from randomly selected rats from each group were stained.

CD20, CD3, and CD68 staining was analyzed using Histoquest**®** software. Digital pictures were taken and 10 high power fields (HPF) per specimen were examined for analysis (original × 400, covering an area of 296 μm × 222 μm) of each graft as previously described [18]. Using Histoquest® software, the number of CD68^+^, CD20^+^, and CD3^+^ cells were counted in relation to all cells within a defined area. Furthermore, we used immunofluorescence to better visualize the distribution of CD20^+^ cell population within the grafts on formalin-fixed, paraffin-embedded materials.

Immunofluorescence staining for CD20 was performed as previously described [20] on 1–2 randomly selected sections from each experimental group. Sections (4 μm) were deparaffinized and rehydrated. Antigen retrieval was performed in a decloaking chamber (Biocare Medical, Pacheco, USA) by treatment in citrate buffer and Antigen Unmasking Solution (Linaris, H-3300, Dossenheim, Germany). Sections were blocked using Superblock Solution (Pierce Technology, 37,515, Rockford, USA). The polyclonal goat anti-rat CD20 antibody (1:100, Santa Cruz, sc-7735, Heidelberg, Germany) was used at 1:100 in PBS overnight. After subsequent washing steps, the tissue was incubated with the donkey anti-goat-FITC antibody (1:500 in PBS, Dianova, 705–095-147, Hamburg, Germany) for 1 h at room temperature. Cell nuclei were stained using Hoechst 33,342 (Molecular Probes H-1399, Waltham, USA) 1:50,000 in PBS for 2 min. at room temperature. Sections were assessed and images taken using a Zeiss observer Z.1 Fluorescence microscope at 20× magnification. Staining specificity of anti-CD20, anti-CD3, and anti-CD68 antibodies was confirmed by anatomical staining pattern of B, and T cells, and monocytes/macrophages respectively in immunofluorescence of rat spleen sections, showing CD20-positive B cell zones and CD3-positive T cell zones in splenic follicles, as well as dispersed distribution of CD68-positive macrophages in the splenic red pulp (Additional file 1 and Additional file 2). A facs co-stain of rat monocyte/macrophage marker CD11b/c (mouse anti-rat CD11b/c-PE, eBiosciences 12–0110-82, San Diego, USA) and CD68 is also presented in the additional files section (Additional file 3). T cells were stained using rabbit anti-rat/hu/ms CD3 (5690, Abcam, Cambridge, UK) and donkey anti-rabbit-Cy5 (Dianova, 711–175-152) as secondary antibody (Additional file1) or donkey anti-rabbit-biotin (Dianova, 711–065-152) and Strep-594 (Dianova, 016–580-084) (Additional file 2). B-cells were stained using mouse anti-rat CD20 (SantaCruz, sc-393,894) and goat anti-mouse(IgM)-biotin (ThermoFisher, 31,804) as a secondary antibody with strep-Cy3 (Dianova, 016–160-084) (Additional file 1) or using goat anti-rat CD20 (SantaCruz, sc-7735) and donkey anti-goat-FITC (Dianova, 705–095-147) (Additional file 2). Macrophages were stained using mouse anti-rat CD68 (BioRad, MCA341GA) and donkey anti-mouse-DyLight 650 (Abcam, ab98797) as a secondary antibody.

### Flow Cytometry

Rat spleen was mechanically macerated to yield single cell suspensions for antibody staining and flow cytometry. Spleen was coarsely chopped using a scalpel, then passed through a 70 μm cell strainer, washed, and then passed through a 30 μm filter. The cell suspension was further separated using ficoll gradient centrifugation. The white cell layer (buffy coat) was collected and used for FACS staining. Cells were blocked using 10% BSA PBS. The following antibodies were used: mouse anti-rat CD11b/c-PE (eBiosciences 12–0110-82, San Diego, USA) and mouse anti-rat CD68 (BioRad, MCA341GA) with secondary antibody donkey anti-mouse-DyLight 650 (Abcam, ab98797).

### Masson Trichrome staining

Renal tissues were fixed in 4% paraformaldehyde and embedded in paraffin, cut into 4-μm thick sections and stained with hematoxylin and eosin (HE) and Masson’s trichrome (MT) staining as follows. First, sections were deparaffinized and rehydrated by treatment with decreasing percentages of ethanol and rinsing in deionized water. Sections were then treated with Bouin’s solution containing Pikrin acid, 5% acetic acid and 10% formaldehyde overnight and then rinsed. This was followed by 5 min. of Weigerts iron-hematoxylin solution in order to stain cell nuclei dark blue. Then Bieberich-Scarlet red acid-fuchsin was applied for 5 min. to stain cytoplasms red. Phosphorus tungsten and phosphorus molybdenic acid was applied for 5 min., followed by Anilin blue solution for 15 min., 1 min of 1% acetic acid, and after rinsing with water, sections were dehydrated using ethanol treatments in increasing concentrations. The morphological changes were examined under a Zeiss Axiostar microscope equipped with a digital camera and analyzed by Metamorphe software (Metamorph 4.6 Universal Imaging Corporation). Depending on the size of the tissue section 10 to 20 images per section (×20 magnification) were captured along the renal cortex, in order to calculate the total percentages of the fibrotic areas for each section.

### Real-time PCR

After homogenization of frozen tissue sections using RNeasy MiniKit® (cat. 74,106, Qiagen, Hilden, Germany) total RNA was extracted, with additional DNase digestion to remove all traces of genomic DNA. Total RNA was reverse transcribed into cDNA: cDNA probes were synthesized in 20 μL reaction volume with 1 μg total RNA, 0.5 μg oligo(dT) primer (Promega, Mannheim, Gemany), 40 units of RNasin (Promega, Mannheim, Germany), 0.5 mM dNTP (Biolabs, Frankfurt am Main, Germany), 4 μL 5× transcription buffer and 200 units of Moloney murine leukemia virus (M-MLV) reverse transcriptase (Promega, Mannheim, Germany) for 1 h at 37 °C. In parallel, no-RT and no-template controls were performed. RT-PCR was performed on ViiA7 detection system in triplicates (Applied Biosystems, Darmstadt, Germany) using QuantiTect SYBR Green PCR Kit (Qiagen, Hilden, Germany). Hypoxanthine-guanine-phosphoribosyl-transferase (HPRT) was used as reference gene. All water controls were negative for target and housekeeper. The sequences of the primers were: rHPRT forw: 5’-CTTTGGTCAAGCAGTACAGCC-3′; rHPRT rev: 5’-TCCGCTGATGACACAAACATGA-3′; rCCL2 forw: 5’-ATGCAGTTAATGCCCCACTC-3′; rCCL2 rev: 5’-TTCCTTATTGGGGTCAGCAC-3′; rCCL5 forw: 5’-CTGCCCCTACTTGTCATGGT-3′; rCCL5 rev: 5’-AGATGAGCCTCACAGCCCTA-3′; rCCR5 forw: 5’-CTATGCCCTTGTTGGGGAGA-3′; rCCR5 rev: 5’-TCCTGTGGACCGGGTATAGA-3′, rCXCL13 forw: 5’-GCAAAAATCAGGCTTCCAGA -3′; rCXCL13 rev: 5’-GGGTCACAGTGCAAAGGAAT-3′; rCCL19 forw: 5’-AGACTGCTGCCTGTCTGTGA-3′; rCCL19 rev: 5’-GCTGGTAGCCCCTTAGTGTG-3′; rCCL20 forw: 5’-CAACTTTGACTGCTGCCTCA-3, rCCL20 rev: 5’-CGGATCTTTTCGACTTCAGG-3; rCCR7 forw: 5’-GGTCATTTTCCAGGTGTGCT-3, rCCR7 rev: 5’-AGTTCCGCACATCCTTCTTG-3; rLymphotoxin-β forw: 5’-TATCAC TGTCCTGGCTGTGC-3′, rLymphotoxin-β rev: 5’-GAGATGCACGAGGGTTTGTT-3′; rCCL21 forw: 5’-ACTGCAGGAAGAATCGAGGA-3′; rCCL21 rev: 5’-TGGACTGTGAACCACTCAGG-3′; rBAFF-R forw: 5’-GTGGGTCTGGTCAGTCTGGT -3′; rBAFF-R rev: 5’-CATTTTCCAGGGACTCTTGG-3′; rBAFF forw: 5’-CTGGAAACTGCCATGCTTCT-3′; rBAFF rev: 5’-TTCGTATAGTCGGCGTGTTG-3′; rIgG forw: 5’-CATTCCCTGCCCCCATC-3′; rIgG rev: 5’-CCGTTCATCTTCCACTCCGT-3′. rCXCL12 forw: 5’-CTGCCGATTCTTTGAGAGCC-3′; rCXCL12 rev: 5’-TTCGGGTCAATGCACACTTG-3′; rCXCR4 forw: 5′- TCTGAGGCGTTTGGTGCT-3′; rCXCR4 rev: 5’-CAGACCCTACTTCTTCGGA-3′. cDNA quantity was determined using a standard curve. Quantity values of target genes were normalized to the house-keeping gene HPRT, and x-fold change of normalized target gene values compared to syngeneic Tx d6 (used as calibrator) was calculated.

### Quantification of donor-specific antibodies (DSA)

Donor (Brown Norway) splenocytes were isolated by macerating spleen through a 100 μm and 40 μm cell strainer, followed by ficoll centrifugation and collection of the white cell layer. Recipient serum was heat-inactivated (56 °C for 30 min.) in order to disable complement factors. Donor splenocytes were incubated with recipient serum for 30 min at 4 °C and then washed. Cells were then stained using either monoclonal mouse anti-rat IgM-PE (eBioscience, 12–0990, San Diego, USA) or polyclonal chicken anti-rat IgG-AlexFluor647 antibody (Invitrogen/Thermo Fischer, A21472, Waltham, USA). As positive controls, we incubated donor splenocytes with heat-inactivated goat or rabbit serum and stained for either goat (donkey anti-goat IgG-DyLight 650, Abcam 96,938, Cambridge, UK) or rabbit (donkey anti-rabbit IgG-Cy5, Dianova, 711–175-152, Hamburg, Germany) antibody. Finally, cells were stained for CD3-FITC (eBioscience 11–0030, San Diego, USA) and measured by flow cytometry. Data is shown for CD3^+^-gated cells, in order to avoid skewing of data by Fc-receptor binding of non-specific antibodies.

### Complement-dependent Cytotoxicity assay (CDC)

Donor splenocytes were isolated as above and resuspended in RPMI1640 Medium (Gibco/Thermo Fischer, Waltham, USA) containing 10% inactivated FCS and 1%Penicillin/Streptomycin. Heat-inactivated recipient serum and donor splenocytes (200,000 cells/well) were incubated at 4 °C for 30 min. Rabbit complement (BAG 7018, Lich, Germany) was added and incubated for 2 h at 24 °C. Goat (DAKO X0907, Hamburg, Germany) or rabbit (DAKO X0902, Hamburg, Germany) serum were used as positive control. Cells were washed and stained with propidium iodide (PI) (Invitrogen/Thermo Fischer, Waltham, USA) to distinguish dead cells and then measured by flow cytometry. Complete lysis was measured using FixPerm (Thermo Fischer, Waltham, USA). Percent cytotoxicity was calculated using the formula: (“PI^+^cells in sample” – “PI^+^cells in medium”)/(“PI^+^cells in FixPerm” – “PI^+^cells in Medium”) ×100.

### Statistical analysis

Values are provided as mean ± SEM. Statistical analysis was performed by the non-parametric Mann-Whitney *U*-test. *p* < 0.05 was considered to be statistically significant.

## Results

### Pronounced rejection and acceleration of chronic interstitial damage under conditions simulating non-adherence

**Fig. 1:**
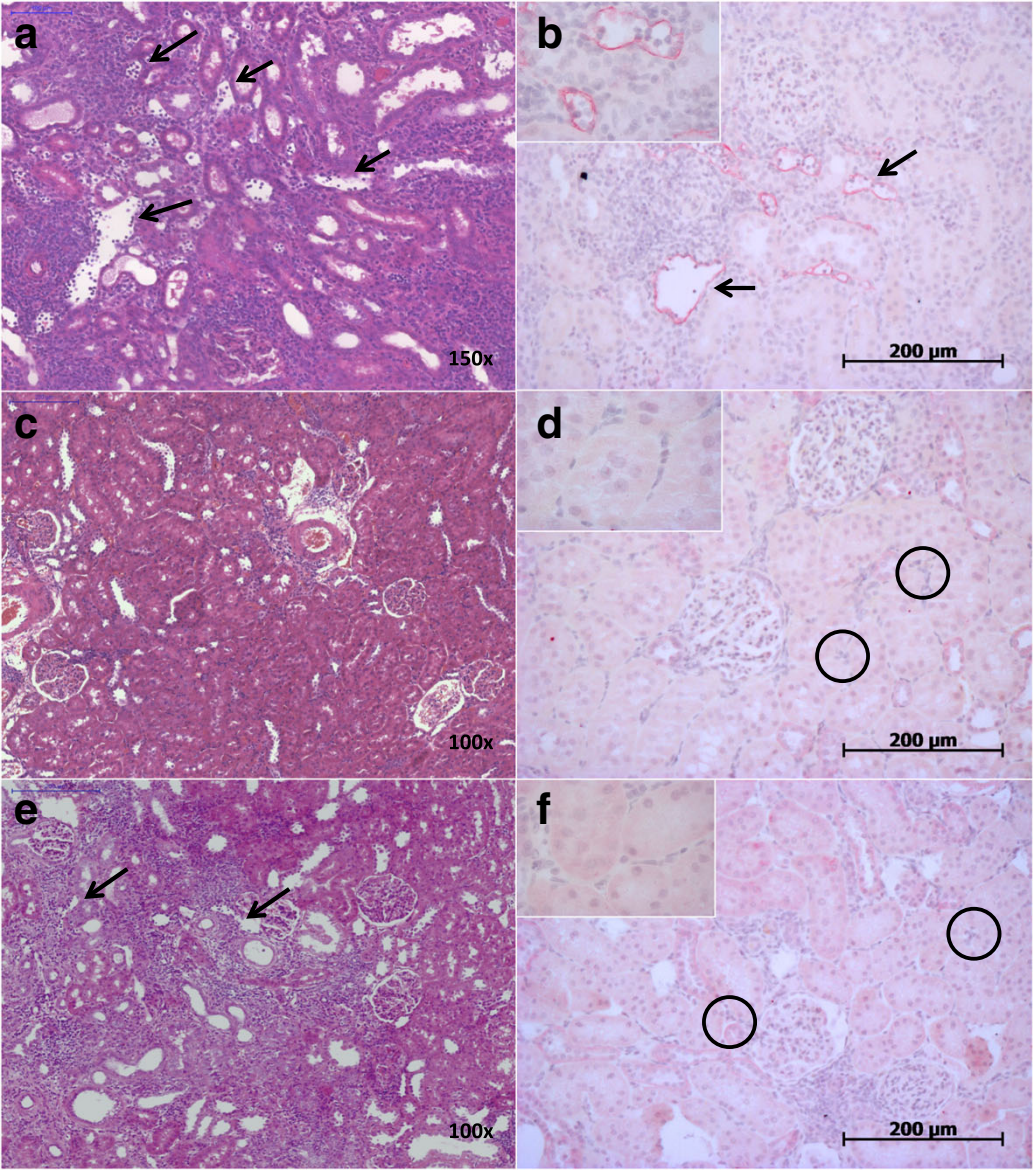
**a-f** Representative renal allograft sections are shown from each group of transplanted rats (*n* = 6–11) (**a-b**) Txd6CyA (**c**-**d**) Txd28CyA, and (**e**-**f**) Txd28CyAalt. The left shows hematoxylin & eosin staining with arrows showing peritubular capillaritis. The right shows anti-rat C4d staining (brown) with arrows pointing out dilated peritubular capillaries containing marginated mononuclear cells and neutrophils and C4d-positivity and black circles showing negative anti-rat C4d staining in peritubular capillaries

**Fig. 2:**
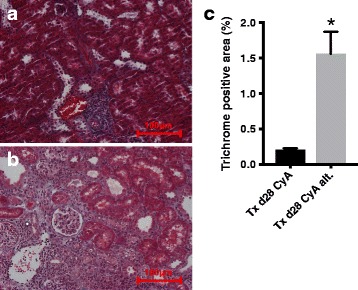
**a-c** Trichrome staining was used to quantify interstitial fibrosis. Representative sections are shown (**a**) Txd28CyA and (**b**) Txd28Cyalt. In (**c**) quantitative analysis of percent trichrome positive area is shown. 6 rats per group were analyzed. The mean and standard error is shown. Significance (Mann-Whitney *U* Test) is shown as * *p* < 0.05 compared to “Txd28CyAalt”

Syngeneic transplantation did not lead to any relevant histological changes (Table 1). Allogeneic transplantation lead to mixed cellular and humoral rejection at day 6 (Txd6CyA). In this group, cellular rejection occurred primarily in the form of tubulitis (Table 1), although 1/11 allografts also had signs of vascular rejection (Banff class 4 IIA). Ten of 11 grafts from this group showed specific features of humoral rejection, including discrete to moderate peritubular capillaritis and C4d-positive staining along peritubular capillaries (Table 1, Fig. 1a-b). After continued daily immunosuppression until day 28 (Txd28CyA), 3/6 grafts showed normal histology, but 3/6 grafts still showed cellular rejection with beginning endothelialitis and predominantly perivascular infiltrates, but without signs of additional humoral rejection (Table 1, Fig. 1c-d). When cyclosporine was only administered on alternating days simulating non-adherence (Txd28CyAalt.), graft histology showed moderate cellular rejection in 5/6 grafts, with endothelialitis and perivascular infiltrates, and 3/6 grafts from this group also showed signs of humoral rejection with ongoing peritubular capillaritis, but negative C4d staining (Table 1, Fig. 1e-f). In addition, trichrome staining showed a significant increase of interstitial fibrosis in the non-adherent group in comparison to the adherent group as shown in Fig. 2a-c (Txd28CyA vs. Txd28CyAalt. *p =* 0.0043) demonstrating an acceleration of chronic interstitial changes induced by intermittent immunosuppression.

**Table 1:** Histopathological classification of allograft rejection in analogy to the Banff classification. Group sizes ranged from 6 to 11 rats per group

Group	No rejection	Class 2 I-II C4d-positive	Class 2 I-II C4d-negative	Class 4 IA	Class 4 IB	Class 4 IIA	Class 4 IIB	Mixed Rejection
Syn Tx d6 (*n* = 8)	8							
Tx d6 CyA (*n* = 11)		10		2	8	1		10
Tx d28 CyA (*n* = 6)	3					3		
Tx d28 CyA alt. (*n* = 6)	1		3			5		3

### Infiltration of inflammatory cell populations

**Fig. 3:**
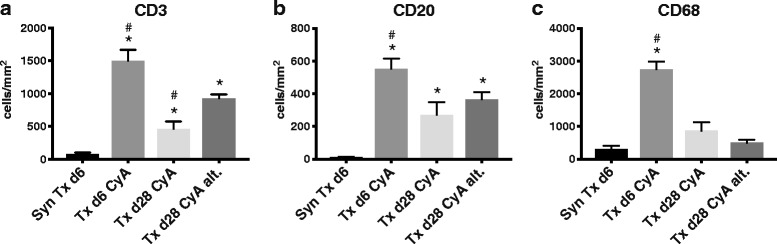
**a-c** Immunohistochemistry was performed on rat kidneys after syngeneic or allogeneic transplantation treated with CyA for 6 or 28 days, or CyA on alternating days until d28. Sections were analyzed using Histoquest® software after staining for rat CD3, CD20, and CD68. The mean number of CD3^+^/CD20^+^/CD68^+^cells/mm^2^ and standard error from at least 6 different animals per group is shown. Significance (Mann-Whitney *U* Test) is shown as * *p* < 0.05 compared to “SynTxd6”, and # *p* < 0.05 compared to “Txd28CyAalt”

Immunohistochemical staining of CD3, CD68, and CD20 in kidney sections showed that, as expected, allogeneic transplantation led to a significant infiltration of CD68^+^ monocytes/macrophages, and CD3^+^ and CD20^+^ lymphocytes by day 6 when compared to syngeneic transplantation (Fig. 3 a-c). When daily cyclosporine treatment was continued until day 28 (Txd28CyA), intra-renal infiltration of all 3 cell populations was strongly reduced in comparison to day 6 after allogeneic transplantation (CD3 *p* = 0.004, CD20 *p* = 0.06, CD68 *p* = 0.002). However, after simulation of “non-adherence” (Txd28CyAalt.), strong CD3^+^ cell infiltration was sustained (*p* = 0.0047 for CD3^+^ cell infiltration in TxCyAd28 vs. Txd28CyAalt.). CD20^+^ cell infiltration in non-adherence was similar to infiltration after daily immunosuppression, but was significantly elevated compared to syngeneic transplantation. Monocyte/macrophage numbers were as low after non-adherence as after syngeneic transplantation.

### Distribution of B cells in clusters after non-adherence

**Fig. 4:**
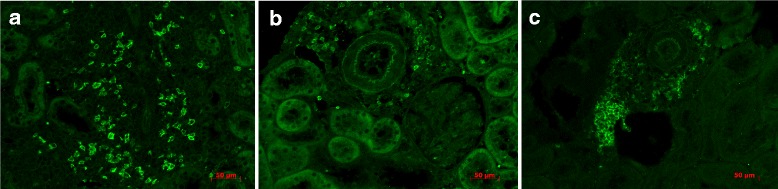
**a-c** Immunofluorescent staining of CD20 in renal allograft was performed. Representative sections from Txd6CyA (**a**), Tx28CyA (**b**), and Txd28CyAalt. (**c**) are shown. Anti-CD20 was labeled with a fitc-conjugated secondary antibody (bright green)

Although the total number of intra-renal CD20^+^ B lymphocytes only showed a slight increase after non-adherence compared to daily immunosuppression, which did not reach statistical significance at this group size, analysis of B cell distribution by immunofluorescent staining showed a remarkable trend towards more organized clusters of B cells. As shown in representative sections in Fig. 4, infiltrating CD20^+^ cells are randomly scattered in allograft tissue at day 6 (Fig. 4a) and moved into a distinctly grouped distribution after continued daily immunosuppression until day 28 (Fig. 4b). When immunosuppression was intermittently omitted in analogy to non-adherence, B cells formed dense clusters as shown in Fig. 4c. Fully matured tertiary lymphoid follicles were not observed at this time-point.

### Chemokines recruit adaptive immunity during non-adherence

**Fig. 5:**
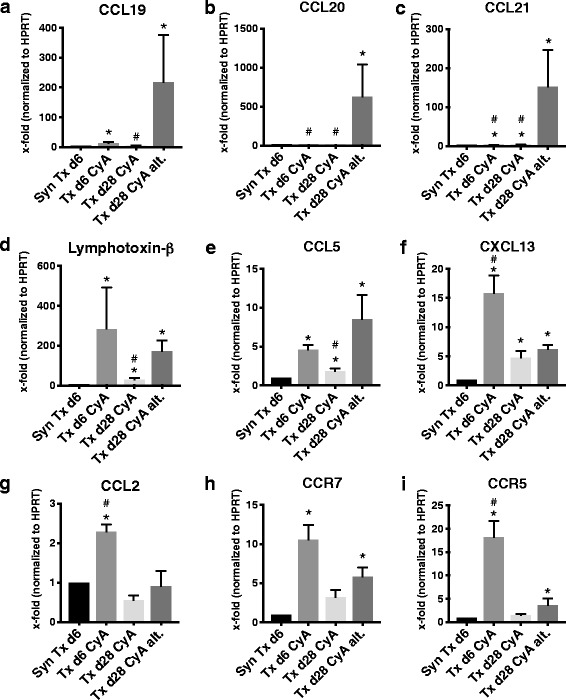
**a-i** Quantitative RT-PCR analysis of chemokine and chemokine receptor expression from renal grafts after syngeneic or allogeneic transplantation treated with CyA for 6 or 28 days, or CyA on alternating days until d28. mRNA expression of target genes was normalized to the house-keeping gene HPRT and x-fold expression in comparison to syngeneic Tx d6 is shown. In (**a**) CCL19, (**b**) CCL20, (**c**) CCL21, (**d**) Lymphotoxin-β, (**e**) CCL5, (**f**) CXCL13, (**g**) CCL2, (**h**) CCR7, and (**i**) CCR5. Data is shown as mean ± SEM. Groups consisted of at least 6 animals. Statistical analysis is shown (Mann-Whitney *U* Test). Significance is shown as *p < 0.05 compared to “SynTxd6”, and # *p <* 0.05 compared to “Txd28CyAalt”

**Fig. 6:**
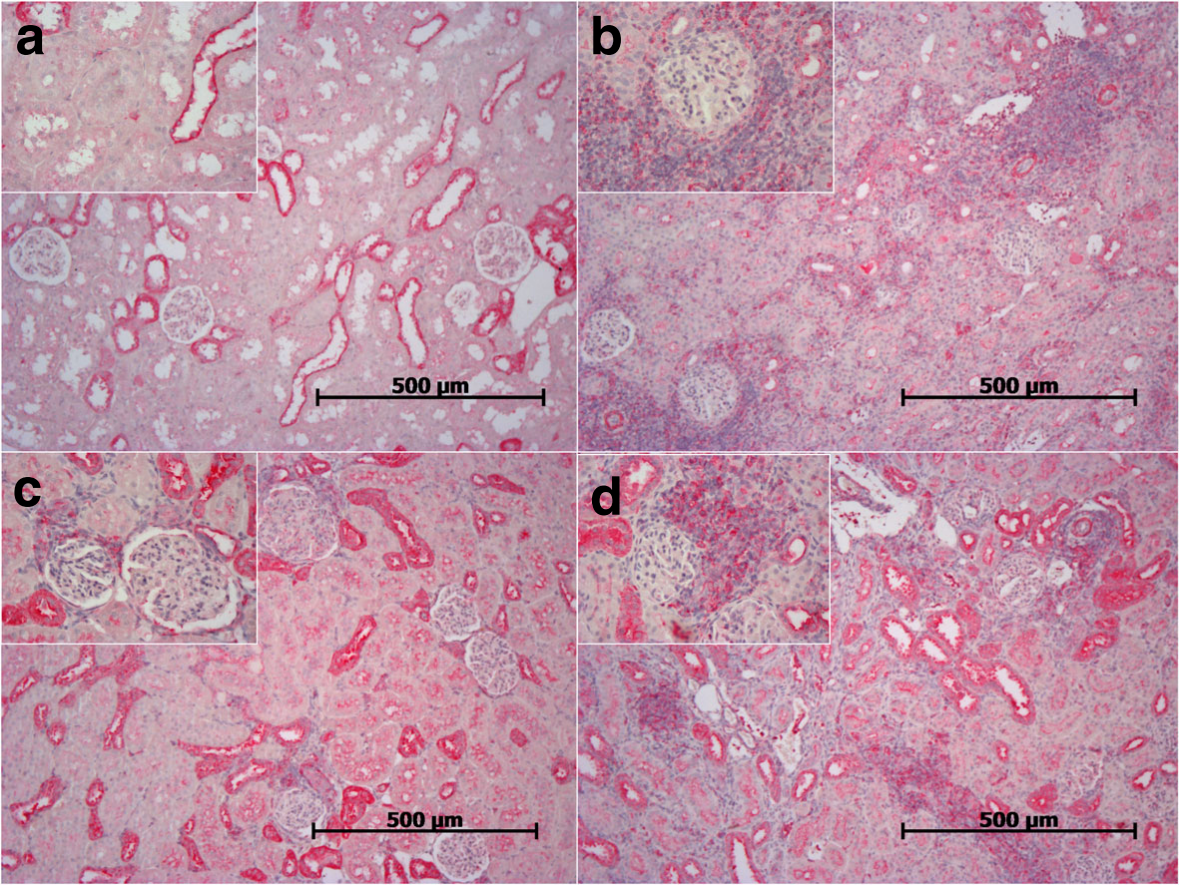
**a-d** CCR-7 expression in renal allografts. Two to three randomly selected sections per group were stained with anti-CCR7 antibody. Representative sections are shown. (**a**) synTxd6 (**b**) Txd6CyA (**c**) Txd28CyA and (**d**) Txd28CyAalt

**Fig. 7:**
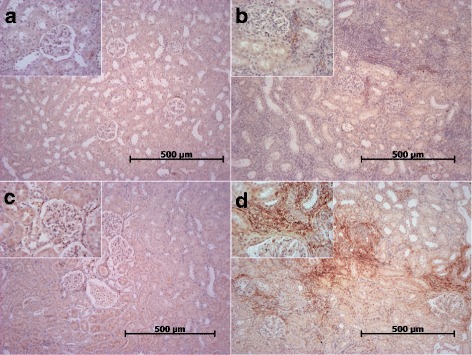
**a-d** CCL21 expression in renal allografts. Two to three randomly selected sections per group were stained with anti-CCL21 antibody. Representative sections are shown. (**a**) synTxd6 (**b**) Txd6CyA (**c**) Txd28CyA and (**d**) Txd28CyAalt

Intra-graft chemokine transcription was compared to infiltrating leukocyte populations. Fittingly, a strong induction of chemokines attracting lymphocytes (CCL19/CCL20/CCL21/Lymphotoxin-β/CCL5), rather than monocytes (CCL2), was seen under conditions simulating non-adherence (Fig. 5); *p-*values for the difference in “non-adherence” compared to standard immunosuppression at day 28 were: *p* = 0.009 for CCL19, *p* = 0.03 for CCL20, *p* = 0.019 for CCL21, *p* = 0.004 for Lymphotoxin-β, *p* = 0.03 for CCL5, and *p* = 0.90 for CCL2. However, an induction of corresponding chemokine receptors CCR7 (CCL19/CCL21 receptor) and CCR5 (CCL5 receptor) was not observed after “non-adherence” (Fig. 5). However, immunohistochemical staining of CCR7 of renal allograft sections revealed positive staining of leukocyte infiltrates, and this was increased in the groups Txd6CyA and Txd28CyAalt. (Fig. 6) compared to synTxd6 and Txd28CyA. In the non-adherence group CCR7-staining of infiltrates was more pronounced than in infiltrates from group Txd6CyA. Notably, tubular epithelial cells were also CCR7-positive, which has previously been reported in the context of renal transplantation [22]. Overall, the intensity of CCR7 staining reflected the pattern of mRNA expression in the different experimental groups. Interestingly, transcription of CCL19, CCL20, and CCL21 was not induced early after transplantation (day 6), but strongly induced after a period of “non-adherence” (Txd6CyA vs. Txd28CyAalt: *p* = 0.0001 for CCL19, *p* = 0.005 for CCL20, *p* = 0.008 for CCL21, Fig. 5). In order to confirm mRNA expression on a protein level, immunohistochemical staining of CCL21 was performed in 2–3 randomly selected allograft sections from each group (Fig. 7). The intensity of CCL21 staining correlated well with PCR data, where expression was low in all groups except the non-adherence group (Fig. 7d). Here, strong CCL21 expression was localized in areas of lymphocyte infiltration. Transcription of the B cell specific chemokine CXCL13 was not significantly induced after “non-adherence”. CXCL12 and its receptor CXCR4 represent an important axis for migration of T, B, and plasma cells, as well as dendritic cells [23], and have been shown to induce dendritic cell rich TLOs in mice [24], however the mRNA expression of CXCR4 and CXCL12 was relatively low. Intra-graft CXCR4 expression was increased in Txd6CyA, but no other group compared to syngeneic Tx d6 (Additional file 4). CXCL12 expression was low and did not differ in the different experimental groups (Additional file 4).

### Non-adherence increases intra-renal B cell activating factor (BAFF) and IgG transcription

**Fig. 8:**
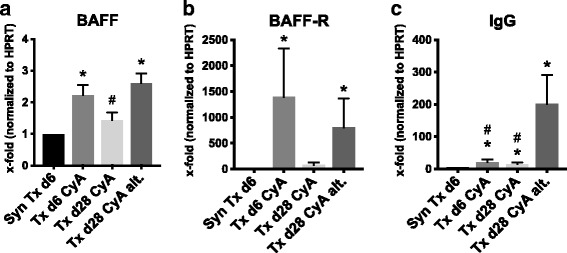
**a-c** Quantitative RT-PCR analysis of BAFF, BAFF receptor and IgG expression from renal grafts after syngeneic or allogeneic transplantation treated with daily CyA for 6 or 28 days, or CyA on alternating days until d28. mRNA expression of target genes was normalized to the house-keeping gene HPRT and x-fold expression in comparison to syngeneic Tx d6 is shown. In (**a**) BAFF, (**b**) BAFF-Receptor, and (**c**) IgG. Data is shown as mean ± SEM. Groups consisted of at least 6 animals. Statistical analysis is shown (Mann-Whitney *U* Test). Significance is shown as * *p* < 0.05 compared to “SynTxd6”, and # *p* < 0.05 compared to “Txd28CyAalt”

Intra-graft BAFF (B cell activating factor belonging to the TNF family) transcription is induced early after allogeneic transplantation (*p* = 0.0003 for Txd6CyA vs. SynTxd6) and is additionally significantly increased in non-adherence compared to standard immunosuppression at day 28 (*p* = 0.026 for Txd28CyAalt. vs. Txd28CyA, Fig. 8a). BAFF-receptor transcription levels are also significantly increased after non-adherence (*p* = 0.0007 compared to control *n* = 6, Fig. 8b). A significant rise of intra-graft Immunoglobulin G (IgG) transcription was observed in the non-adherence group in comparison to all other groups (Txd28CyAalt. vs. Txd28CyA *p* = 0.004, vs. Txd6 *p* = 0.0028, vs. SynTxd6 *p* = 0.0007, Fig. 8c).

### Development of de novo donor-specific antibodies

**Fig. 9:**
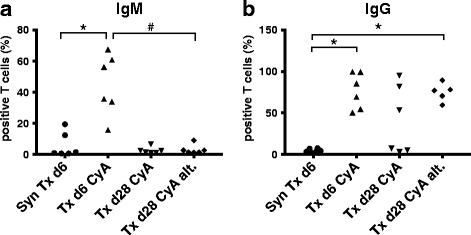
**a-b** Detection of donor-specific antibodies from rats after syngeneic or allogeneic kidney transplantation treated with daily CyA for 6 or 28 days, or CyA on alternating days until d28. Donor splenocytes were incubated with recipient serum and then stained with (**a**) anti-rat IgM-antibody or (**b**) anti-rat IgG-antibody in duplicates and measured by flow cytometry. Percentages of IgG or IgM-positive cells are shown. Data is shown as single data points of each group on a scatter plot. Groups consisted of 5–6 animals. Statistical analysis is shown (Mann-Whitney *U* Test). Significance is shown as * *p* < 0.05 compared to “SynTxd6”, # *p* < 0.05 compared to “Txd28CyAalt”

**Fig. 10:**
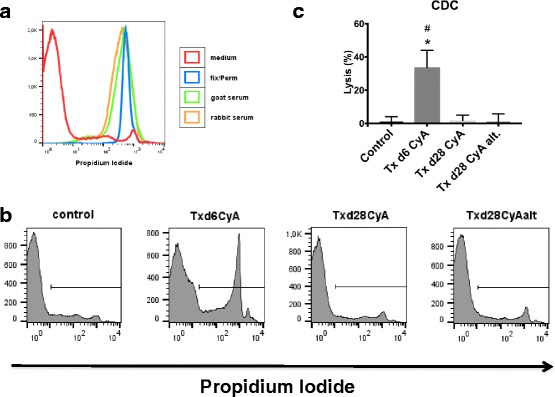
Complement-dependent-cytotoxicity of donor-specific antibodies from rats after syngeneic or allogeneic kidney transplantation treated with daily CyA for 6 or 28 days, or CyA on alternating days until d28. Donor splenocytes were incubated with recipient serum and rabbit complement. Dead cells were stained with propidium iodide (PI) and measured by flow cytometry. (**a**) shows representative histograms of PI-staining for negative control (cell medium, shown in red) and positive controls (Fix/perm, shown in blue; goat serum shown in green; rabbit serum shown in orange). (**b**) shows representative histograms of PI-staining for control (Lew d0), Txd6CyA, Txd28CyA, and Txd28CyAalt. (**c**) shows quantitative analysis as percent lysis (*n* = 3–4 per group). Significance (Mann-Whitney *U* Test) is shown as * *p* < 0.05 compared to control (Lewis rat serum pre-Tx d0), # *p* < 0.05 compared to “Txd28CyAalt”

To test for DSA, recipient serum was incubated with donor splenocytes and stained for rat IgM (immunoglobulin M) and IgG (immunoglobulin G). Goat and rabbit serum were expected to contain xenoantibodies and were used as positive controls showing robust and titratable positive signals for the anti-rat IgG and IgM FACS stain (data not shown) and also complement-dependent cytotoxicity (Fig. 10a). Recipient sera after syngeneic transplantation did not show positive anti-rat IgG or IgM staining on donor splenocytes (Fig. 9a-b). However, allogeneic transplantation led to the appearance of high levels of donor-specific IgM and IgG early after transplantation (day 6) (*p* = 0.004 for Txd6CyA vs. SynTxd6 for IgM and *p* = 0.002 for IgG). As expected, donor-specific IgM disappeared by day 28 (Fig. 9a). IgG levels also markedly decreased by day 28 under daily immunosuppression in 3/6 subjects, but remained elevated in another 3/6 (Fig. 9b, Txd28CyA). A highly relevant observation was, that the same 3/6 rats from the group showing virtually no DSA also lacked histological signs of rejection; in contrast, the remaining 3/6 rats which did show histological signs of rejection (Table 1), also displayed measurable amounts of DSA in serum. In the non-adherence group, donor-specific IgG was elevated in all measured samples (*p* = 0.004 for SynTxd6 vs. Txd28CyAalt.), and this corresponded with histological signs of C4d-negative ABMR in 3/6 animals in this group.

In order to rule out any binding of non-specific immunoglobulins, we incubated recipient sera, which had previously shown IgG-binding on donor splenocytes (Txd6CyA) with recipient Lewis rat splenocytes (data not shown). There was no staining of rat-IgG or IgM on Lewis splenocytes, confirming that the observed anti-rat IgG are donor-specific. Similarly, Lewis rat serum from day 0 did not show any specific binding of anti-rat IgG on BN splenocytes (data not shown).

Using a complement-dependent cytotoxicity assay, we found that only DSA produced early after transplantation (Txd6CyA) activated complement-mediated cytotoxicity (CDC) (Fig. 10, *p* = 0.03 for Txd6CyA vs. Lewis Serum d0), while no cytotoxicity was observed after 28 days of daily or alternating CyA treatment, (*p* = 0.03 for Txd6CyA vs. Txd28CyAalt.), eventhough DSA were detected in this group by flow cytometry (Fig. 9b). These results are consistent with the pattern of C4d-staining in the different groups, which showed positive C4d staining at day 6 after allogeneic Tx, but not at day 28 after daily or intermittent immunosuppression (Fig. 1).

## Discussion

Since non-adherence to immunosuppressive therapy is strongly associated with donor-specific antibodies and accelerated graft failure, our aim was to utilize a rat model of non-adherence in order to study the immunological mechanisms underlying chronic allograft injury.

In our model, MHC-mismatched rat strains were used for allogeneic renal transplantation. Acute humoral and cellular rejection was observed at day 6. As the animals were not pre-sensitized, the immunological risk was considered low, and no induction therapy was used. When immunosuppression was continued, rejection subsided in some animals, but not all, probably because the dose of cyclosporine used (5 mg/kg) was relatively low and no induction therapy or steroid was administered on top. Under daily cyclosporine administration, normal histology or mild cellular rejection was seen at day 28. In contrast, the non-adherence group suffered from a significant increase in the rate of cellular rejection with additional features of acute humoral rejection – illustrated by initiation of peritubular capillaritis. Our model intended to show mechanisms of early chronic parenchymal changes. Such changes were indeed induced in our model of non-adherence, as demonstrated by a significant increase in interstitial fibrosis.

While cellular infiltration was minimal after syngeneic transplantation, high numbers of inflammatory cells were seen early after allogeneic transplantation (day 6) with monocytes dominating the infiltrate. The numbers of monocytes, T cells and B cells declined when standard immunosuppression with daily CyA was continued. However, under conditions simulating non-adherence, T cell infiltration did not resolve as quickly as under daily immunosuppression. Meanwhile, B cell numbers remained elevated in comparison to syngeneic transplantation and monocyte numbers declined to a level similar to that after syngeneic transplantation. In line with these results, Hueso et al. previously showed that early interstitial fibrosis/tubular atrophy (IF/TA) and reduced graft survival are associated with increased infiltration of T and B cells in human renal transplant biopsies [25].

The changes seen in the pattern of intra-renal chemokine transcription mirrored the changes in the composition of the cellular infiltrate, with chemokines attracting T and B lymphocytes, such as CCL19, CCL20, CCL21, CCL5 and lymphotoxin-β, increased in non-adherence.

Interestingly, CCL19, CCL20, and CCL21, were much more strongly induced in the setting of non-adherence than during the initial inflammatory reaction early after Tx (d6). CCL19, CCL21, and their receptor CCR7 are known to regulate homing and co-localization of dendritic cells and naïve T cells in lymphoid organs and are essential to T cell sensitization and the formation of an adaptive immune response [26]. However, they have also been implicated in the formation of tertiary lymphoid organs (TLO) in the context of chronic inflammation [24, 27, 28]. These structures have also been found in transplant organs and are associated with a poorer outcome [14, 29]. In a murine model of kidney transplantation, the fusion protein CCL19-IgG, which interferes with normal CCL19-CCR7 signaling, was found to strongly reduce graft rejection [30]. In our model of non-adherence, chemokines associated with TLO formation are strongly expressed, and this is accompanied with the formation of dense lymphocyte aggregates.

Another factor that has been associated with chronic inflammation and formation of B cell rich TLO is B cell activating factor (BAFF) [31]. BAFF is an activation, maturation and survival factor for B cells, expressed by lymph node stromal cells, neutrophils, macrophages, monocytes, dendritic cells and T cells [32]. A pathogenetic role for BAFF has been suggested for several autoimmune diseases, including Sjögren Syndrome, systemic lupus erythematosus, and multiple sclerosis [33–35]. In the context of renal transplantation, higher serum levels of BAFF are associated with donor-specific antibodies [36], blood cell-bound BAFF with worse renal graft function, and intra-graft BAFF expression is associated with ABMR and IF/TA [37]. We now show that BAFF transcription is increased within the graft during non-adherence. In line with this, IgG transcription levels are also increased during non-adherence. In fact, the non-adherence group was the only group that showed intra-renal IgG transcription, demonstrating that local intra-graft antibody formation exclusively occurred after prolonged suboptimal immunosuppression.

Although, fully developed TLO structures were not yet observed in our experimental setting at the analyzed time-points, the enhanced organization of B lymphocytes into dense clusters during our simulation of non-adherence together with the increased expression of TLO-associated chemokines CCL19, CCL20, CCL21, and BAFF may be indicative of early steps in the formation of these highly organized structures. Furthermore, the appearance of intra-graft IgG transcription maybe linked to the development of an organized local adaptive immune response. Although our experiments cannot differentiate plasma cell infiltration from local differentiation from precursor cells, a possible explanation maybe that BAFF activates intra-renal B cells, which mature into antibody-secreting plasma cells within the graft. Similarly, our experiments cannot rule out that increased IgG transcription is due to turnover of local B cell populations in non-adherence, but evidence for local antibody production by plasma cells in chronic rejection has been provided by Thaunat et al. [14]. There is also evidence for clonal expansion of B cells inside grafts [38]. No conclusions can be drawn as to the specificity or diversity of the IgG produced in our non-adherence model, and further experiments will be needed to establish the source and specificity of intra-graft IgG production. Others have shown however, that locally and systemically produced antibodies differ in diversity and timing with more diverse HLA (human leukocyte antigen) specificities being generated from intra-graft antibody production [14, 39].

In our model, de novo DSA were detected in rat serum under conditions mimicking non-adherence to therapy. This corresponds to data from studies of renal transplant patients [3, 10, 11]. Antibodies were shown to be donor-specific, since binding of recipient Lewis rat splenocytes did not occur. Although our experiments could not specifically identify anti-MHC antibodies, endothelial non-MHC targets for these antibodies were unlikely since splenocytes were used in our assay. IgM and IgG DSA were detected early after transplantation (day 6), following a kinetic previously described for rat humoral responses after immunization [40, 41]. At the later timepoint, we saw a clear induction of a secondary humoral response, where IgM was no longer detected, while IgG levels remained elevated. We interpreted this as a sign that Ig class switching was completed, and that sensitization to donor antigens and initial DSA production takes place very early. Our results suggest that activated B cells and plasma cells are then armed and ready for antibody production, and are kept in check by appropriate immunosuppression with CNI. Analysis of CDC showed that DSA were cytotoxic early after Tx (d6), but not after non-adherence or daily CyA (d28), which was consistent with the pattern of C4d-staining observed in the different groups. Our experiments do not offer a specific mechanism to explain this phenomenon, but one possibility is that the different immunosuppressive treatments and durations result in a switch in the IgG-subclass generated and thereby also determine the phenotype of ABMR, eg. C4d-positive vs. C4d-negative, in line with recently published observations of DSA IgG subclasses and rejection phenotypes in transplant patients [42]. In this study of renal transplant patients, chronic ABMR was associated with the non-complement activating IgG subclass IgG4 in humans, whereas acute ABMR was associated with the complement-fixing IgG3 [42].

Under-immunosuppression in a clinical setting may be due to non-adherence to therapy or to inter-individual variations in responsiveness to CNI, since CNI therapy is monitored using serum concentration, not functional tests. Considering this, even when optimal adherence to therapy is achieved, a group of patients may still effectively be “under-immunosuppressed” and at risk of chronic rejection.

While there is a lot of data showing the deleterious effects of DSA on graft outcome, there is an ongoing debate over which DSA are clinically relevant, or more precisely, what features of DSA, such as MFI (mean fluorescence intensity), complement-fixing capacity or IgG-subclass, are linked to deleterious outcomes and require treatment [42–44]. Our model offers an ideal framework for deciphering such critical issues.

Several animal models have been established in which preformed DSA are induced using pre-transplant immunization [45, 46]. Since in the majority of cases, DSA are de novo DSA and not DSA from pre-sensitization, our model - with reliable histological and serological entities of acute antibody mediated rejection during non-adherence - more closely resembles this highly prevalent group of patients.

## Conclusion

In this study, we established and characterized a rat model of CNI under-immunosuppression in analogy to non-adherence to immunosuppressive therapy after allogeneic kidney transplantation. In this model, non-adherence led to mixed cellular and humoral rejections. This study shows that during prolonged under-immunosuppression, lymphocytic infiltrates take up an organized form within the graft. This is promoted by factors also associated with formation of secondary and tertiary lymphoid organs. Furthermore, intra-renal IgG mRNA synthesis was induced after prolonged under-immunosuppression. These intra-renal changes are accompanied by systemic production of donor-specific antibodies after non-adherence. The contribution of organized lymphocytic infiltrates to chronic allograft injury needs to be addressed in further studies.

## Additional files

Additional file 1: *anti-CD20 and anti-CD3 immunofluorescence costaining of rat spleen follicles*. Anti-CD20-positive B cells are in the B cell zone of splenic follicles (yellow), and anti-CD3-positive T cells in the T cell zone of the splenic follicles (red), also shown are proliferating Ki67-positive cells in splenic follicles (green). (PDF 3198 kb)

Additional file 2: *anti-CD20, anti-CD3, and anti-CD68 immunofluorescence costaining of rat spleen follicle*. (A) shows anti-CD20-positive B cells in the B cell zone of a splenic follicle (green), anti-CD3-positive T cells in T cell zone of splenic follicle (red), and CD68-positive macrophages (yellow) in the splenic red pulp, also shown in (B) without costaining. (PDF 588 kb)

Additional file 3: *anti-CD11b/c and anti-CD68 FACS costain of rat splenocytes*. (A) shows unstained cells, (B) shows anti-CD11b/c-PE antibody only, and (C) shows anti-anti-CD11b/c-PE antibody and anti-CD68-APC antibody co-stain of rat splenocytes. (PDF 82 kb)

Additional file 4: *CXCR4 and CXCL12 expression.* This figure shows quantitative RT-PCR analysis of chemokine and chemokine receptor expression from renal grafts after syngeneic or allogeneic transplantation treated with CyA for 6 or 28 days, or CyA on alternating days until d28. mRNA expression of target genes was normalized to the house-keeping gene HPRT and x-fold expression in comparison to syngeneic Tx d6 is shown. (A) CXCR4 (B) CXCL12. Data is shown as mean ± SEM. Groups consisted of at least 6 animals. Statistical analysis is shown (Mann-Whitney *U* Test). Significance is shown as *p < 0.05 compared to “SynTxd6”, and # *p <* 0.05 compared to “Txd28CyAalt”. (PDF 24 kb)

## Abbreviations

ABMR: Antibody-mediated rejection
BAFF: B cell activating factor belonging to the TNF family
BAFF-R: BAFF-receptor
BN: Brown Norway rat
CCL: CC chemokine ligand
CCR: CC chemokine receptor
CD: Cluster of differentiation
CDC: Complement dependent cytotoxicity
CNI: Calcineurin inhibitor
CXCL: CXC chemokine
CXCR: CXC chemokine receptor
CyA: Cyclosporine A
DSA: Donor-specific antibody
HLA: Human leukocyte antigen
IF/TA: Interstitial fibrosis and tubular atrophy
IgG: Immunoglobulin G
IgM: Immunoglobulin M
LEW: Lewis rat
MFI: Mean fluorescence intensity
MHC: Major histocompatibility complex
RT-PCR: Real-time polymerase chain reaction
SLE: Systemic lupus erythematosus
TLO: Tertiary lymphoid organ
Tx: Transplantation
