# Supplementary figures and images for: Renal allograft rejection, lymphocyte infiltration, and de novo donor-specific antibodies in a novel model of non-adherence to immunosuppressive therapy
Source: BMC Immunol. 2017 Dec 19;18:52. doi: 10.1186/s12865-017-0236-6 (PMC5735914; doi:10.1186/s12865-017-0236-6)

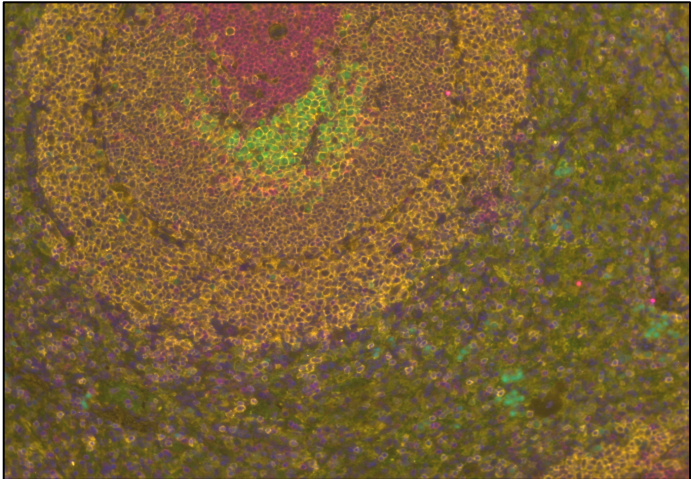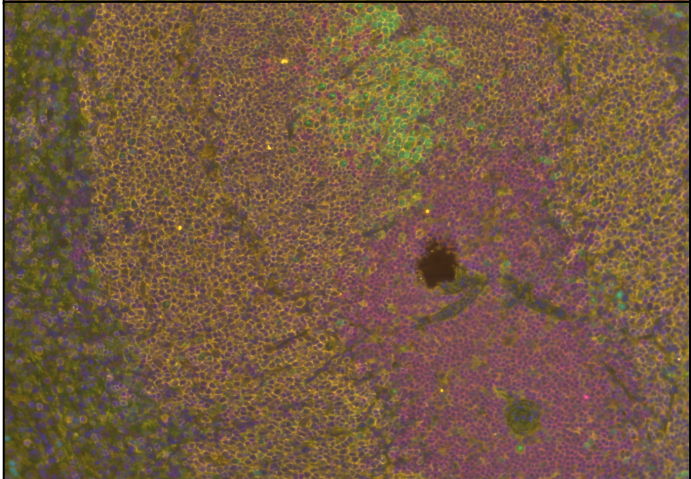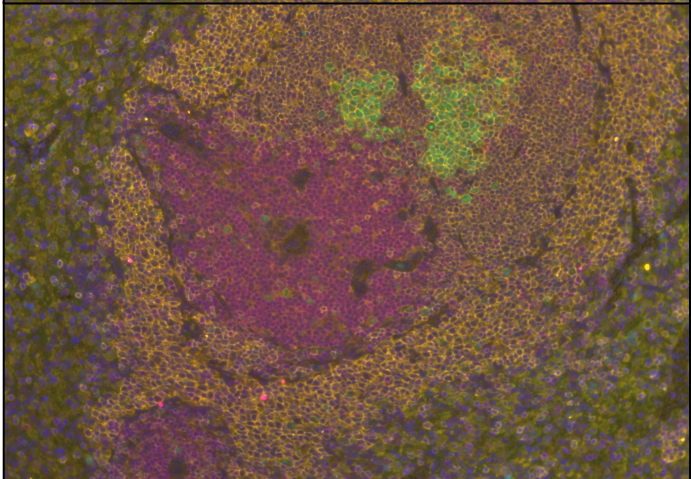

Supplement: Supplementary file 1 — anti-CD20 and anti-CD3 immunofluorescence costaining of rat spleen follicles. Anti-CD20-positive B cells are in the B cell zone of splenic follicles (yellow), and anti-CD3-positive T cells in the T cell zone of the splenic follicles (red), also shown are proliferating Ki67-positive cells in splenic follicles (green). (PDF 3198 kb) [file 12865_2017_236_MOESM1_ESM.pdf]

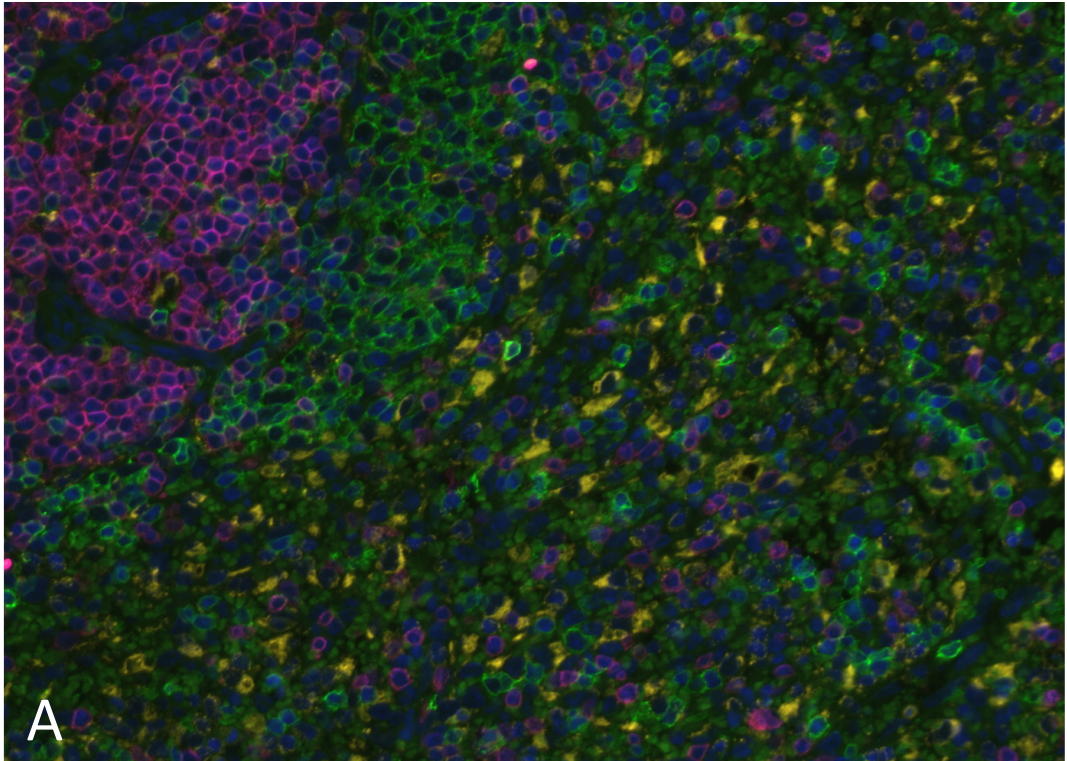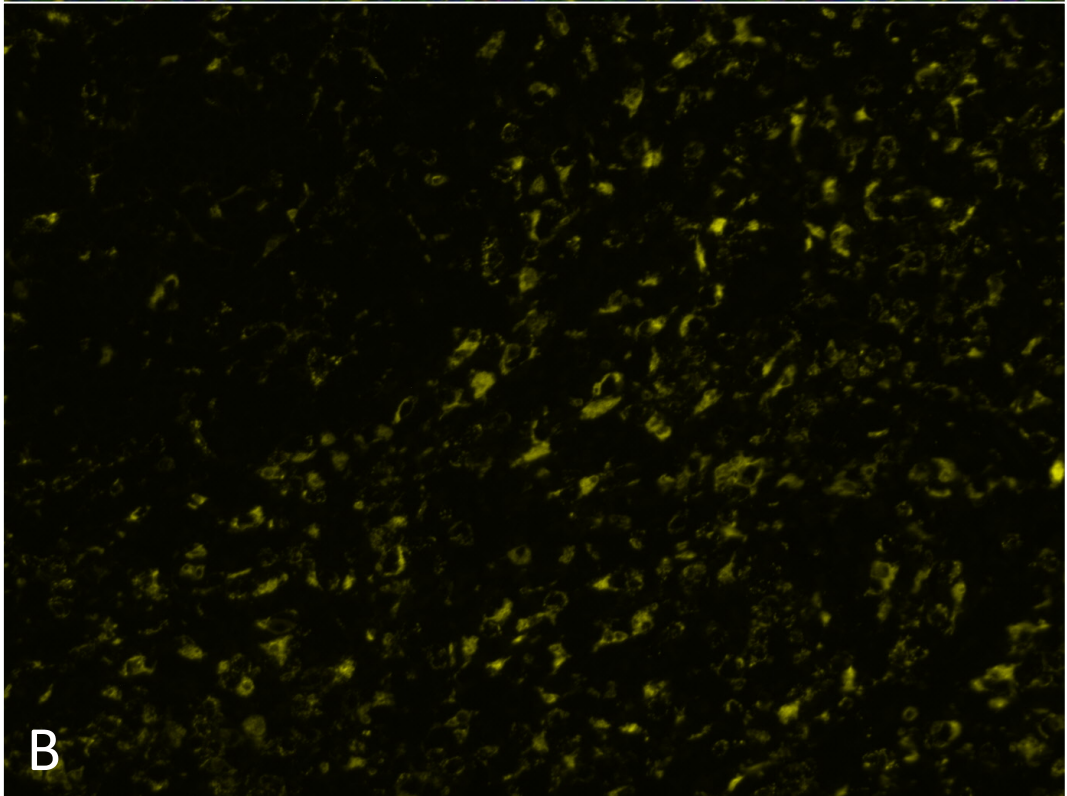

Supplement: Supplementary file 2 — anti-CD20, anti-CD3, and anti-CD68 immunofluorescence costaining of rat spleen follicle. (A) shows anti-CD20-positive B cells in the B cell zone of a splenic follicle (green), anti-CD3-positive T cells in T cell zone of splenic follicle (red), and CD68-positive macrophages (yellow) in the splenic red pulp, also shown in (B) without costaining. (PDF 588 kb) [file 12865_2017_236_MOESM2_ESM.pdf]

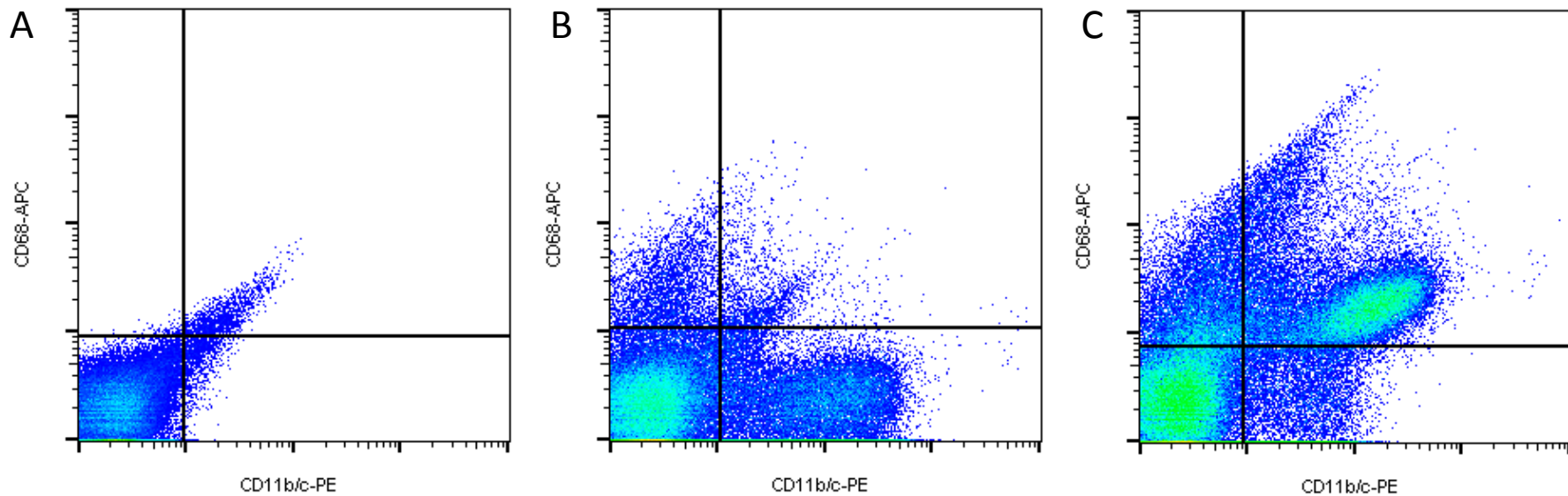

- A) Unstained rat splenocytes
- B) Anti-CD11b/c-PE only
- C) Anti-CD68-APC and anti-CD11b/c-PE

Supplement: Supplementary file 3 — anti-CD11b/c and anti-CD68 FACS costain of rat splenocytes. (A) shows unstained cells, (B) shows anti-CD11b/c-PE antibody only, and (C) shows anti-anti-CD11b/c-PE antibody and anti-CD68-APC antibody co-stain of rat splenocytes. (PDF 82 kb) [file 12865_2017_236_MOESM3_ESM.pdf]

**A****CXCR4**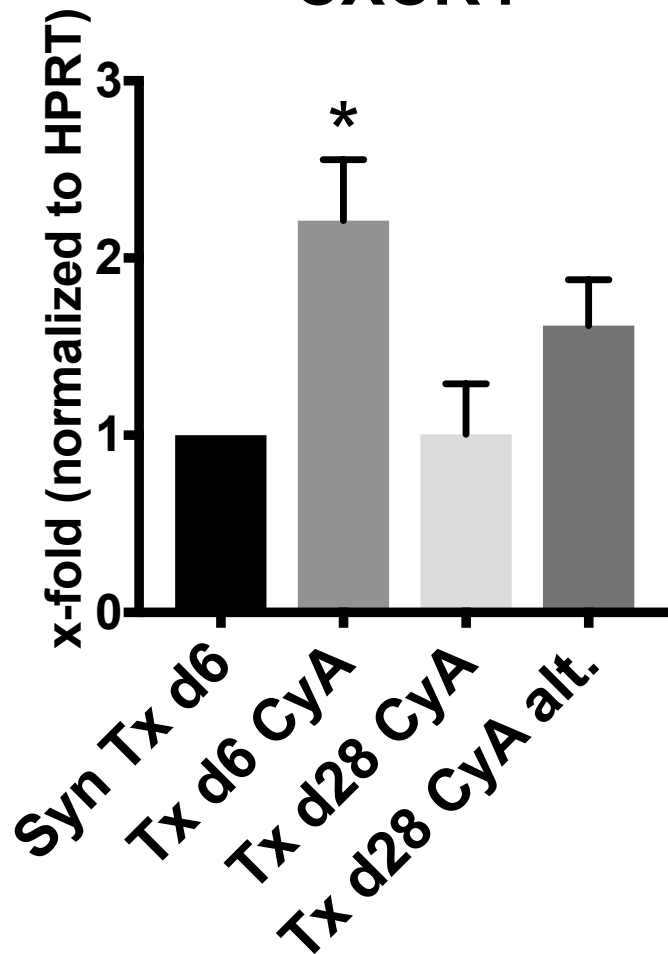**B****CXCL12**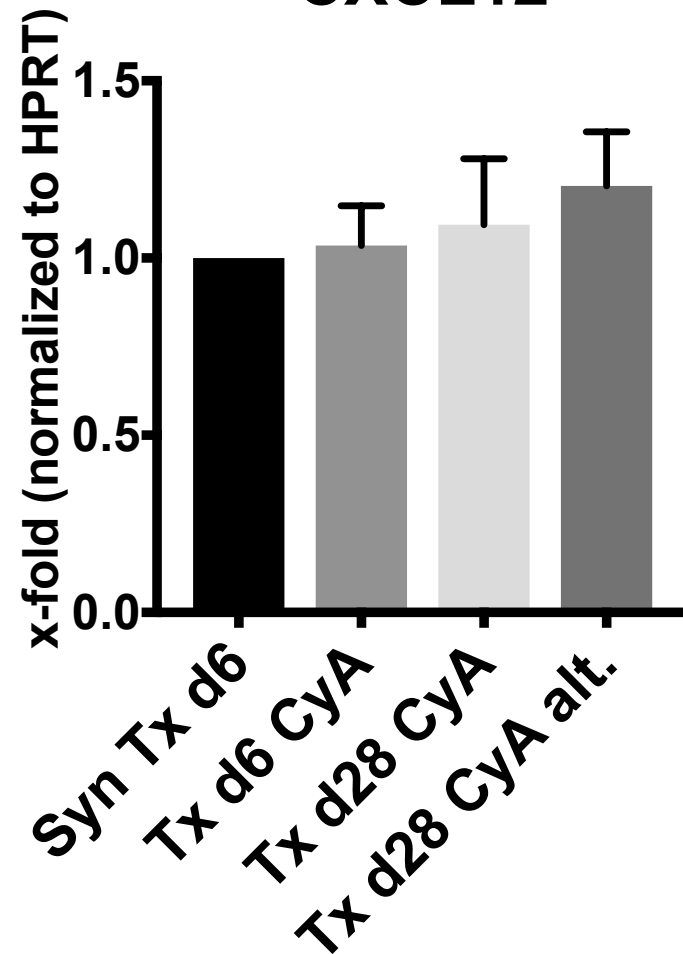

Supplement: Supplementary file 4 — CXCR4 and CXCL12 expression. This figure shows quantitative RT-PCR analysis of chemokine and chemokine receptor expression from renal grafts after syngeneic or allogeneic transplantation treated with CyA for 6 or 28 days, or CyA on alternating days until d28. mRNA expression of target genes was normalized to the house-keeping gene HPRT and x-fold expression in comparison to syngeneic Tx d6 is shown. (A) CXCR4 (B) CXCL12. Data is shown as mean ± SEM. Groups consisted of at least 6 animals. Statistical analysis is shown (Mann-Whitney U Test). Significance is shown as *p < 0.05 compared to “SynTxd6”, and # p < 0.05 compared to “Txd28CyAalt”. (PDF 24 kb) [file 12865_2017_236_MOESM4_ESM.pdf]
